# Lynch II syndrome: a case report

**DOI:** 10.1186/1471-2318-11-S1-A58

**Published:** 2011-08-24

**Authors:** B Scotto, E Spera, G Merola, D Di Simone

**Affiliations:** 1 Department of Surgical, Anesthesiologic and Emergency Sciences. University “Federico II”, Naples , Italy

## Background

Lynch syndrome is an autosomal dominant cancer susceptibility syndrome that accounts for approximately 2–4% of all colorectal cancers (CRCs) and is caused by germline mutations of mismatch repair (MMR) genes [1-4]. It is characterized by an early onset of predominantly CRC and endometrial cancer (EC) as well as cancers of the stomach, small bowel, ureter and renal pelvis.

## Materials and methods

We report a case of a patient 60 years old affected by synchronous primary cancers of the sigma and stomach diagnosed by TC. The patient had previously been operated on for the right hemicolectomy for adenocarcinoma, and hysterectomy for cancer. During hospitalization the patient has undergone surgery on the left hemicolectomy and a total gastrectomy. The patient underwent chemotherapy. Currently the patient is receiving chemotherapy for local recurrence.

## Results

As a result of genetic study, the patient was found to be the first to have this mutation.

## Conclusions

Surgery remains the front-line therapy for HNPCC. There is an ongoing controversy over the benefit of 5-fluorouracil-based adjuvant therapies for HNPCC-related colorectal tumors, particularly those in stages I and II. After reporting a null finding from their randomized controlled trial of aspirin (ASA) to prevent against the colorectal neoplasia of Lynch Syndrome, Burn and colleagues have recently reported new data, representing a longer follow-up period than reported in the initial NEJM paper. These new data demonstrate a reduced incidence in Lynch Syndrome patients who were exposed to at least four years of high-dose aspirin, with a satisfactory risk profile. These results have been widely covered in the media; future studies will look at modifying (lowering) the dose (to reduce risk associated with the high dosage of ASA).

## References

[B1] Fishel R The human mutator gene homolog MSH2 and its association with hereditary nonpolyposis colon cancer Cell 1993 75 1027 38 10.1016/0092-8674(93)90546-3 8252616

[B2] Bronner CE Mutation in the DNA mismatch repair gene homologue hMLH1 is associated with hereditary non-polyposis colon cancer Nature 1994 368 258 61 10.1038/368258a0 8145827

[B3] Miyaki M Germline mutation of MSH6 as the cause of hereditary nonpolyposis colorectal cancer Nat Genet 1997 17 271 2 10.1038/ng1197-271 9354786

[B4] Nicolaides NC Papadopoulos Mutations of two PMS homologues in hereditary nonpolyposis colon cancer Nature 1994 371 75 80 10.1038/371075a0 8072530

[B5] Renda A Relazione Biennale SIC Multiple Primary Malignant 2009 Springer- Verlag Italia

